# Paradoxical Association of Smoking With In‐Hospital Mortality Among Patients Admitted With Acute Ischemic Stroke

**DOI:** 10.1161/JAHA.113.000171

**Published:** 2013-06-21

**Authors:** Syed F. Ali, Eric E. Smith, Deepak L. Bhatt, Gregg C. Fonarow, Lee H. Schwamm

**Affiliations:** 1 Department of Neurology, Massachusetts General Hospital/Harvard Medical School, Boston, MA (S.F.A., L.H.S.); 2 Calgary Stroke Program, University of Calgary, Alberta, Canada (E.E.S.); 3 Brigham and Women's Hospital and VA Boston Healthcare System, Boston, MA (D.L.B.); 4 Division of Cardiology, Ronald Reagan‐UCLA Medical Center, Los Angeles, CA (G.C.F.)

**Keywords:** cerebrovascular disease, embolic stroke, mortality, thrombolysis

## Abstract

**Background:**

Compared to those who never smoked, a paradoxical effect of smoking on reducing mortality in patients admitted with myocardial ischemia has been reported. We sought to determine if this effect was present in patients hospitalized with ischemic stroke.

**Methods and Results:**

Using the local Get with the Guidelines‐Stroke registry, we analyzed 4305 consecutively admitted ischemic stroke patients (March 2002–December 2011). The sample was divided into smokers versus nonsmokers. The main outcome of interest was the overall inpatient mortality. Compared to nonsmokers, tobacco smokers were younger, more frequently male and presented with fewer stroke risk factors such as hypertension, hyperlipidemia, diabetes, coronary artery disease, and atrial fibrillation. Smokers also had a lower average NIH Stroke Scale (NIHSS) and fewer received tissue plasminogen activator (tPA). Patients in both groups had similar adherence to early antithrombotics, dysphagia screening prior to oral intake, and deep vein thrombosis (DVT) prophylaxis. Smoking was associated with lower all‐cause in‐hospital mortality (6.6% versus 12.4%; unadjusted OR 0.46; CI [0.34 to 0.63]; *P*<0.001). In multivariable analysis, adjusted for age, gender, ethnicity, hypertension, diabetes mellitus, hyperlipidemia, CAD, atrial fibrillation, NIHSS, and tPA, smoking remained independently associated with lower mortality (adjusted OR 0.64; CI [0.42 to 0.96]; *P*=0.03).

**Conclusions:**

Similar to myocardial ischemia, smoking was independently associated with lower inpatient mortality in acute ischemic stroke. This effect may be due to tobacco‐induced changes in cerebrovascular vasoreactivity, or may be due in part to residual confounding. Larger, multicenter studies are needed to confirm the finding and the effect on 30‐day and 1‐year mortality.

## Introduction

The term “smoker's paradox” was first introduced into scientific discourse more than 25 years ago following observations that smokers (in comparison to nonsmokers) experience decreased mortality following an acute myocardial infarction (AMI).^1–4^ The smoker's paradox is one of several reported paradoxes in the cardiovascular literature. Other major paradoxes assessing the association of a vascular risk factor with health outcomes after a cardiovascular event include the obesity paradox and the gender paradox. The obesity paradox identifies a decrease in morbidity and mortality with increasing body mass index (BMI) while the gender paradox identifies the purportedly protective association with male gender.^5–9^ This report focuses attention on the interaction between smoking status and outcomes after acute ischemic stroke−the so‐called smoker's paradox−and highlights the challenges of distinguishing residual and unmeasured confounding from true associations in cardiovascular disease epidemiology.

While the smoker's paradox in cardiovascular disease patients has been partly explained by younger age of onset and fewer coexisting high‐risk features in patients with AMI who are current smokers, a small number of studies have shown that the residual lower mortality risk persists despite comprehensive covariate adjustment.^2,10–13^ Aune et al^14^ published a systematic review in 2011 reporting that the paradox was observed in some studies of AMI patients in the prethrombolytic and thrombolytic era, whereas no studies of a contemporary population with acute coronary syndrome have found evidence for such a paradox. Furthermore, some angiographic studies have demonstrated that coronary artery occlusion in smokers is predominantly caused by thrombosis and thus may have a better response to spontaneous or therapeutic thrombolysis.^15–19^

The etiology of myocardial infarction and acute ischemic stroke is similar in terms of occlusion leading to ischemia. Similar risk factors predispose patients to ischemic stroke as that of myocardial infarction. Despite the plethora of data exploring smokers' post‐AMI survival advantage, the role of smoking in the short‐term prognosis after acute ischemic stroke remains to be clarified.

Saver et al analyzed the National Institute of Neurological Disorders and Stroke (NINDS) tissue plasminogen activator (tPA) stroke trials data which suggested that recent smokers with acute ischemic stroke who received intravenous (IV) thrombolysis experienced a better early outcome than nonsmokers. Furthermore, the study suggested that fewer thrombolysis‐treated smokers may exhibit global disability at 90 days and that thrombolysis‐treated smokers may have better survival rates at 1 year than thrombolysis‐treated nonsmokers.^20^

These and other observations motivated us to perform a retrospective analysis of our acute ischemic stroke (AIS) patients' database and to evaluate if a similar association of smoking and short‐term outcomes was present. The objective of this study was to examine the associations between demographic and clinical characteristics, presentation, in‐hospital treatment, and mortality among patients with and without active cigarette smoking (any cigarette use in the past year) who were hospitalized with AIS.

## Methods

### Patients Population

Using our hospital's Get with the Guidelines‐Stroke (GWTG‐Stroke) registry, we analyzed patients consecutively admitted with AIS. GWTG‐Stroke is an ongoing, voluntary, continuous registry and performance improvement initiative. It collects patient‐level data on characteristics, diagnostic testing, treatments, adherence to quality measures, and in‐hospital outcomes for those hospitalized with AIS. Trained hospital personnel ascertain consecutive patients admitted with stroke by either prospective clinical identification, retrospective identification using International Classification of Diseases (ICD)‐9 discharge codes, or a combination of both. Prospective identification includes regular surveillance of emergency department records (ie, presenting symptoms and chief complaints), ward census logs, and/or neurological consultations. The eligibility of each acute stroke is confirmed at chart review before abstraction. After abstraction by trained personnel at the hospital, deidentified patient data are entered into the GWTG‐Stroke database using a Web‐based patient management tool (PMT, Outcome). Data abstracted included patient demographics, medical history and comorbidities, calendar year, initial head computerized tomography findings, in‐hospital treatment and events, discharge treatment and counseling, mortality, and discharge destination.

After excluding patients with possible transient ischemic attacks (TIAs) and stroke mimics, our cohort consisted of 4305 AIS patients admitted from March 2002 until December 2011. Data on demographics, comorbidities, NIHSS at the time of admission, in‐hospital interventions, length of stay, and mortality were collected and analyzed.

### Definitions

#### Smoking

The study population was divided into 2 groups based on their smoking status. Information about smoking status was abstracted from the medical record by a trained abstractor and classified according to the GWTG‐Stroke coding instructions which are derived from the Medicare definition of smoking status. The 2 groups consisted of current smokers (any cigarette use within the year preceding the stroke) versus nonsmokers (either never smokers or those who had been abstinent for >1 year prior to stroke). The data do not distinguish between the 2 subtypes of nonsmokers.

#### GWTG‐stroke in‐hospital measures

Inpatient management including performance of dysphagia screening, use of early antithrombotics and DVT/PE prophylaxis were all recorded at the patient level according to GWTG‐Stroke definitions. The absolute rates of these interventions are listed in Table 1 among all patients admitted, without exclusions for ineligibility.

**Table 1.** Baseline Characteristics, In‐Hospital Treatments, and Mortality of Patients Admitted With Acute Ischemic Stroke According to Smoking Status

	Smokers (n=703)	Nonsmokers (n=3602)	*P* Value
Age, y	59.6±13.8	70.8±14.8	<0.001
Gender (male)	422 (60.0%)	1923 (53.4%)	<0.001
Race (white)	641 (91.2%)	3151 (87.5%)	0.01
Medical history			
HTN	387 (55.0%)	2525 (70.1%)	<0.001
DM	122 (17.4%)	883 (24.5%)	<0.001
HL	246 (35.0%)	1497 (41.6%)	<0.001
CAD	101 (14.4%)	848 (23.5%)	<0.001
Previous stroke/TIA	80 (11.4%)	482 (13.4%)	0.15
A Fib	60 (8.5%)	876 (24.3%)	<0.001
PVD	46 (6.5%)	171 (4.7%)	0.05
NIHSS (n=657 vs 3343)	4 (2 to 11)	5 (2 to 13)	<0.001
Median (IV tPA cohort)	13	13	0.23
tPA (all types)	100 (14.2%)	683 (19.0%)	<0.001
tPA at MGH	39 (5.5%)	252 (7.0%)	0.16
tPA at OSH	61 (8.7%)	431 (12.0%)	0.01
IA therapy at MGH	30 (6.6%)	213 (8.1%)	0.28
GWTG measure adherence			
Early antithrombotics	640/684 (93.6%)	3183/3446 (92.4%)	0.07
Dysphagia screening	439/634 (69.2%)	2366/3241 (73.0%)	0.08
DVT/PE prophylaxis*	525/532 (98.7%)	2796/2849 (98.1%)	0.67
Complications			
Pneumonia*	84/685 (12.3%)	333/3517 (9.5%)	0.03
UTI*	12/270 (4.4%)	143/1869 (7.7%)	0.06
Length of stay (days)	5.0±4.3	4.8±4.2	0.22
Unable to ambulate at D/C	85 (12.1%)	612 (17.0%)	0.002
Discharge disposition			
Home	336 (47.8%)	1370 (38.0%)	<0.001
IRF	289 (41.1%)	1496 (41.5%)	
SNF	32 (4.6%)	259 (7.2%)	
Expired	46 (6.5%)	477 (13.2%)	
In‐hospital mortality	46 (6.5%)	477 (13.2%)	<0.001

HTN indicates hypertension; DM, diabetes mellitus; HL, hyperlipidemia; CAD, coronary artery disease; TIA, transient ischemic attack; A Fib, atrial fibrillation; PVD, peripheral vascular disease; tPA, tissue plasminogen activator; MGH, Massachusetts General Hospital; OSH, Outside Hospital; GWTG, Get with the Guidelines; DVT, deep vein thrombosis; D/C, discharge; NIHSS, NIH Stroke Score; IV, intravenous; IA, intraarterial; UTI, urinary tract infection; IRF, inpatient rehabilitation facility; SNF, skilled nursing facility.

* Variables with rates of missing >10% (UTI, 49.5%; DVT/PE, 12.5%).

#### Outcome

The primary outcome of interest for this study was in‐hospital mortality, which was defined as patients who expired during their stay at the hospital or were discharged to hospice for end‐of‐life care. We had in‐hospital mortality data for all 4305 patients included in the study.

### Statistical Analysis

The sample was divided into the 2 aforementioned groups and frequencies with percentages were generated for the dichotomous variables (gender, race, comorbidities, in‐hospital interventions, and mortality). Values for age were expressed as mean±SD and that for NIHSS as median with interquartile ranges. Pearson chi‐square was employed for categorical variables to compare the baseline characteristics of the 2 groups. For continuous variables, we used independent *t*‐test for age and Wilcoxon rank sum test for comparative analysis of NIHSS between smokers and nonsmokers. Unadjusted univariate OR with 95% CIs were computed for all variables, but reported for only those variables that were significant in univariate testing. All tests of statistical significance were 2‐tailed and were considered to be significant at a 0.05 level of statistical significance, unless otherwise specified.

Multivariable logistic regression analysis was employed to determine independent associations between in‐hospital mortality and all covariables significant in univariate analysis (*P*<0.1). Variables that reflect processes of care during hospitalization or complications were not included in the model. To avoid any bias associated with variable selection, we repeated the multivariable model with all covariates.

We also carried out secondary analyses by restricting our study cohort to evaluate if the association of smoking with in‐hospital mortality persists in (1) patients with first ever stroke, excluding those with a prior history of stroke, (2) patients without a prior history of CAD, (3) patients who received IV tPA, and (4) patients stratified by age at admission (≤60 and >60 years). Statistical analyses were performed using the software SPSS, version 20.0.

## Results

### Characteristics of the Study Population

We indentified a total of 4305 AIS patients, out of whom 703 (16.3%) were current smokers. Mean age was observed to be 69.1±15.2 years; 54.5% of patients were male and 88.1% were white. Vascular comorbidities were frequent with hypertension being the most common in 67.6% of AIS patients, hyperlipidemia (HL) in 40.5%, diabetes mellitus (DM) in 23.3%, CAD in 22.0% and peripheral vascular disease (PVD) in 5.0%. Atrial fibrillation (Afib) was seen in 21.7% of AIS patients.

A total of 244 (5.7%) of patients with AIS had a prior episode of stroke. Eighteen percent of patients were treated with IV, while 6% received intra‐arterial tPA. Most of the patients admitted were given early antithrombotics (92.6%), underwent dysphagia screening (72.4%) before commencement of oral intake and had DVT/PE prophylaxis (98.4%) within 48 hours of arrival. Common complications recorded were in‐hospital pneumonia in 10.0% (417/4202) and urinary tract infection (UTI) in 7.2% (155/2139).

As expected, the groups of smokers and nonsmokers differed significantly in many characteristics, as shown in Table 1. Smokers, on average, were ≈10 years younger and were more often males. They were also more frequently white with a higher percentage of peripheral vascular disease. Smokers had fewer risk factors for stroke as compared to nonsmokers. They were significantly less likely to have a history of hypertension (HT), DM, HL, CAD, and Afib. Smokers presented with a 1‐point lower median NIHSS (4 versus 5) which was significant when compared to nonsmokers. A greater percentage of nonsmokers received thrombolysis (IV or intraarterial). The absolute rates of dysphagia screening, antithrombotic use and DVT prevention were similar for the 2 groups. Smokers were more likely to experience hospital‐acquired pneumonia, while UTI was more common in nonsmokers.

### In‐Hospital Mortality Rates

Smoking was associated with lower all‐cause in‐hospital mortality. Only 6.5% of smokers died during their stay as compared with 13.2% of nonsmokers. In univariate analysis, smoking was associated with lower unadjusted OR for death of 0.46 (95% CI of 0.34 to 0.63 [*P*<0.001]). Smokers also had more favorable discharge destination, with a greater percentage going to home or to an inpatient rehabilitation facility.

Univariate analysis also demonstrated that age was strongly related to in‐hospital mortality. The mean age±standard deviation of the patients with in‐hospital mortality was significantly higher compared to the patients who survived (67.9±15.1 versus 77.1±12.9; *P*<0.001). The results of univariate analysis of in‐hospital mortality rates, according to the baseline characteristics, are shown in Table 2. Other positive predictors of mortality included female gender, white race, and presence of HT, CAD, Afib, PVD, higher NIHSS, and in‐hospital pneumonia. Intervention with IV tPA and smoking were the only 2 factors associated with lower mortality on univariate analysis.

**Table 2.** Factors Significantly Associated With In‐Hospital Mortality in Univariate Analysis and Multivariable Logistics Regression Model

	Unadjusted OR	95% Confidence Interval	Adjusted OR	95% Confidence Interval
Age (per year)	1.05	(1.04 to 1.06)	1.03	(0.43 to 0.96)
Gender (female)	1.30	(1.08 to 1.56)	—	—
Race (white)	1.43	(1.04 to 1.96)	1.55	(1.04 to 2.32)
Comorbidities				
****Hypertension	1.36	(1.10 to 1.66)	—	—
****CAD	1.92	(1.57 to 2.34)	1.70	(1.30 to 2.21)
****A Fib	2.69	(2.22 to 3.26)	—	—
****PVD	1.47	(1.02 to 2.13)	—	—
NIHSS	1.20	(1.19 to 1.22)	1.21	(1.19 to 1.23)
IV tPA	0.51	(0.41 to 0.62)	0.71	(0.55 to 0.93)
Smoking	0.46	(0.34 to 0.63)	0.64	(0.42 to 0.96)
Pneumonia	2.62	(2.04 to 3.36)	—	—

OR indicates odds ratio; CAD, coronary artery disease; A Fib, atrial fibrillation; PVD, peripheral vascular disease; NIHSS, NIH Stroke Score; IV, intravenous; tPA, tissue plasminogen activator.

All significant univariate predictors of in‐hospital mortality were included in a logistic regression model in order to examine their independent association with in‐hospital mortality. Older age, history of cardiovascular disease, white race, and higher NIHSS at presentation remained significantly associated with higher of mortality, while smoking and IV tPA use were associated with lower in‐hospital mortality. A significant change in the rates of smoking was observed over the study period. It increased from 17.4% in 2001 to 22.0% in 2005 and then subsequently dropped to 11.0% in 2011 (*P* value for trend <0.001). In‐hospital mortality varied from 11.5% in 2003 to 14.5% in 2011. Since the rates of smoking varied over the study period, we included a calendar year term in the final model. The adjusted OR for in‐hospital mortality associated with smoking was 0.64 (0.42 to 0.96; *P*=0.03). A repeat analysis using all covariates did not change the adjusted OR for smoking 0.647 (0.43 to 0.96).

### Secondary Analyses

#### First ever stroke

The favorable association of smoking with lower in‐hospital mortality persisted in the sub‐group of patients with first ever index stroke. When a total of 244 patients with prior ischemic stroke were excluded from the analysis (28 smokers, 216 nonsmokers) the observed unadjusted OR remained significant for the association between smoking and mortality (OR 0.45 [0.33 to 0.62]; *P*<0.001). In hospital mortality was 6.4% (n=43) for smokers as compared to 13.1% (n=445) for nonsmokers (*P*<0.001).

#### Cohort without coronary artery disease

Another model consisting of patients without any history of CAD (n=3356) was created to account for any confounding effect of CAD on mortality (n=3356; 602 smokers, 2754 nonsmokers). In‐hospital mortality was 5.6% (n=34) for smokers as compared to 11.5 % (n=316) for nonsmokers (*P*<0.001). Similar results were seen with smoking in patients without CAD (unadjusted OR 0.46 [0.32 to 0.67]; *P*<0.001).

#### Thrombolysis

Out of the 4305 patients included in this study, 783 received IV tPA of which 14.2% (100) were smokers. As shown in Table 1, nonsmokers were more likely to receive IV tPA as compared to smokers. Unadjusted in‐hospital mortality among smokers was significantly lower in thrombolysed patients as well, when compared to nonsmokers (8.0% versus 20.6%; *P*=0.003). On multivariable analysis, even a stronger association was observed with OR 0.33 ([0.16 to 0.71]; *P*=0.004). Positive predictors of mortality in this sub‐group were similar to those in the total cohort and included age (OR 1.06 [1.04 to 1.08]), HT (OR 1.84 [1.21 to 2.80]), CAD (OR 2.54 [1.74 to 3.70]), Afib (OR 2.32 [1.60 to 3.37]), DM (OR 1.51 [0.99 to 2.30]), increasing NIHSS (OR 1.20 per point [1.15 to 1.24]), and development of hospital‐acquired pneumonia (OR 1.68 [1.02 to 2.76]). The only factor associated with lower odds of mortality was smoking. In the cohort of patients who did not receive IV tPA, smoking was still significantly associated with lower mortality, with OR of 0.52 (0.37 to 0.73; *P*<0.001).

#### Different age strata

Since smokers with AIS were significantly younger than nonsmokers, we divided the study population into 2 cohorts based on the median age of smokers (≈60 years). In total there were 1206 patients with age ≤60 years and of these 29.6% (n=357) were smokers. In‐hospital mortality was lower for smokers versus nonsmokers ≤60 years (3.4% versus 6.2%; *P*=0.04) with an OR 0.52 (0.28 to 0.99; *P*=0.046) and in those >60 years (9.8% versus 15.4%, *P*<0.001) with an OR 0.60 (0.41 to 0.87; *P*<0.001).

## Discussion

This study, based on the analysis of all consecutively admitted AIS patients in our major regional stroke center over the past 9 years, demonstrated that smoking is associated with lower in‐hospital mortality after AIS. This finding persisted even after extensive adjustment for measured covariates and temporal trends. These findings extend the “smokers paradox,” which has been previously described for acute coronary syndromes and acute heart failure, to patients hospitalized with AIS, and provide important insights into the relationship of smoking to the age of patients at stroke onset and other demographic and clinical characteristics.

Cigarette smoking is a major risk factor for AIS. Prior studies estimate that smoking nearly doubles the risk of ischemic stroke. Consistent with this risk, patients who were current cigarette smokers in this study presented with stroke at substantially younger ages compared to current nonsmokers. They were also less likely to have other traditional risk factors for stroke. These baseline differences have been reported in the vast majority of previous large‐scale studies examining the smokers' paradox, especially in acute coronary syndrome (ACS) patients.^16,21–23^

Differences in health outcomes at discharge in this study by smoking status may reflect differences in the characteristics of patients with AIS by smoking status. In previous studies, older age had been consistently regarded as the most important factor influencing early prognosis after AIS.^24–25^ In our study, cigarette smokers were 10 years younger compared to nonsmokers, and age‐related differences contributed to the unadjusted survival differences. Cigarette smokers were also most likely to be males. An independent influence of gender on early mortality after AIS has been well documented by Smith et al^25^ in a cohort of 274 988 ischemic stroke patients. Despite the adjustment for age and gender, the reduced mortality associated with smoking persisted. In the literature exploring the effect of smoking on mortality after myocardial infarction, the paradox does not hold true when comparing longer‐term mortality in smokers who quit versus smokers who continued smoking after the index event.^26^ The latter group had a significantly higher mortality, supporting the notion that smoking has deleterious effects in the cardiovascular system. This fact demonstrates that “paradoxes” like this do not prove that a risk factor may also be in some ways protective, but simply identify an association. It is just as likely that among smokers there is a fundamentally different set of pathophysiologic mechanisms contributing to stroke risk and that comparison between the groups therefore remains confounded despite measured covariate adjustment. This distinction requires a large cohort of smokers in whom the quantitative smoking exposure is well characterized, and rates of adverse events are measured both in‐hospital and longitudinally.

Cigarette smoking has been associated by Vlietstra et al^27^ with less extensive and less severe CAD in a cohort of 15 298 patients with established CAD, who were enrolled in the Coronary Artery Surgery Study. Despite the lack of similar data for AIS patients, lower NIHSS observed for smokers may suggest that this finding of decreased severity of the index event holds true for stroke patients as well. In multivariable logistic regression, we included NIHSS as a covariate to adjust for difference in severity on presentation of the 2 groups. Smokers were still observed to have a better prognosis after accounting for NIHSS.

Smoking has been associated with increased hematocrit, platelet activation and aggregation, vasoconstriction, increased circulating levels of fibrinogen, thrombin generation, and impaired endogenous fibrinolytic capacity.^28–30^ As a result, the pathogenesis of vascular occlusions may be more thrombogenic than atherogenic in smokers, particularly those with fewer biomarkers of traditional atherosclerosis risk factors, whereas in nonsmokers, occlusion may be more frequently due to rupture or ulceration of atheromatous plaque with formation of platelet‐rich clot. All of this may reflect a greater susceptibility of cerebral thrombi to spontaneous or therapeutic thrombolysis in smokers, an effect which was observed in our study reflected by lower OR for mortality among smoking in the cohort of patients treated with IV tPA.

Alternatively, it may be that the abrupt cessation of smoking during AIS improves outcomes among smokers, or that medication used to assist in abstinence, such as nicotine replacement, may have some protective role in hospitalized patients with stroke.

Chronic changes in vasomotor tone and episodic hypoxia might lead to ischemic preconditioning in smokers, as well as the development of improved small vessel cerebral collaterals and better cerebral perfusion. These and other changes to brain metabolism might limit the initial injury and influence stroke progression and mortality. These avenues warrant further investigation.

This apparent smoker's paradox in AIS patients should not be interpreted as a benefit of cigarette smoking. The hazardous effects of smoking are manifested as the occurrence of stroke in patients years earlier than might otherwise have occurred. It is likely that our models have been unable to completely control for these differences in age of onset, despite our best attempts in the reported sensitivity analyses. Intensive efforts to encourage smoking cessation as a primary and secondary preventive measure for stroke should remain a very high priority. Effective smoking prevention and cessation methods should be implemented as vigorously as other guideline‐recommended therapies for the prevention of stroke. In fact, the smoker's paradox might just be an apparent one; on further dissection, smokers are getting milder strokes years earlier than they might otherwise experience cerebral ischemia, and there is no evidence that early milder strokes are in anyway protective of later, more disabling age‐appropriate strokes. This apparent paradox deserves much more attention.

Our present analysis of smoking and in‐hospital mortality has some important limitations. We used the GWTG‐Stroke definition of “smoking,” which is derived from Medicare reporting and quality programs that define smokers as those who have used cigarettes in the past year. This is due to the high rates of recidivism seen in this cohort. Therefore, our cohort of smokers is heterogeneous and includes current heavy smokers, recent light smokers, and individuals who are not currently smoking but have done so in the past year. The data could be missing some baseline clinical variables and in‐hospital complications, so we cannot exclude the possibility that unmeasured or residual confounding may explain some or all of our findings. Because smoking dose is not usually in the medical record and therefore we do not have a quantification of smoking exposure (ie, “smoking dose”), our ability to assess correlations between smoking and the age of stroke onset or presence of polyvascular disease is limited. This may contribute to our inability to distinguish a true protective effect from the more likely residual confounding. Smokers may be more likely to have small vessel stroke than cardioembolic or other sub‐types, as compared to nonsmokers, and this may be related to stroke outcomes. Because the stroke subtype is not captured in the database analyzed for this study, we are unable to adjust for this finding. Patients reporting of smoking status may not have been reliable in all cases. No quantitative measures were conducted to evaluate the biologic effects of smoking in patients with AIS such as thrombotic factors, endothelial function, and systemic inflammatory markers. Larger multicenter studies are needed to confirm the finding, investigate a possible difference in stroke sub‐type between the 2 groups, and assess if the mortality benefit persists at 30 days and 1 year post discharge.
